# Chronic radiation-associated dysphagia in oropharyngeal cancer survivors: Towards age-adjusted dose constraints for deglutitive muscles

**DOI:** 10.1016/j.ctro.2019.06.005

**Published:** 2019-06-15

**Authors:** MD Anderson Head and Neck Cancer Symptom Working Group, Kaitlin M. Christopherson, Alokananda Ghosh, Abdallah Sherif Radwan Mohamed, Mona Kamal, G. Brandon Gunn, Timothy Dale, Jayashree Kalpathy-Cramer, Jay Messer, Adam S. Garden, Hesham Elhalawani, Steven J. Frank, Jan Lewin, William H. Morrison, Jack Phan, Neil Gross, Renata Ferrarotto, Randal S. Weber, David I. Rosenthal, Stephen Y. Lai, Katherine Hutcheson, Clifton David Fuller, Spatial-Non-spatial Multi-Dimensional Analysis of Radiotherapy Treatment/Toxicity Team (SMART3), Abdallah Sherif Radwan Mohamed, G. Elisabeta (Liz) Marai, Guadalupe Canahuate, David M. Vock, David Fuller

**Affiliations:** a Department of Radiation Oncology, The University of Texas MD Anderson Cancer Center, Houston, TX, USA; b Department of Biostatistics and Data Science, University of Texas School of Public Health, Houston, TX, USA; c Department of Clinical Oncology, University of Alexandria, Alexandria, Egypt; d Department of Emergency Medicine, The University of Texas MD Anderson Cancer Center, Houston, TX, USA; e Baylor College of Medicine, Houston, TX, USA; f Athinoula A. Martinos Center for Biomedical Imaging, Massachusetts General Hospital, Charlestown, MA, USA; g Department of Radiation Oncology, The University of Texas Medical Branch, Galveston, TX, USA; h Department of Head and Neck Surgery, The University of Texas MD Anderson Cancer Center, Houston, TX, USA; i Department of Thoracic & Head and Neck Oncology, The University of Texas MD Anderson Cancer Center, Houston, TX, USA; j Medical Physics Program, The University of Texas Graduate School of Biomedical Sciences, Houston, TX, USA; a Department of Radiation Oncology, The University of Texas MD Anderson Cancer Center, Houston, TX, USA; b Department of Clinical Oncology, University of Alexandria, Alexandria, Egypt; c Department of Computer Science, University of Illinois at Chicago, Chicago, IL, USA; d Department of Electrical and Computer Engineering, The University of Iowa, Iowa City, IA, USA; e Medical Physics Program, The University of Texas Graduate School of Biomedical Sciences, Houston, TX, USA; f Athinoula A. Martinos Center for Biomedical Imaging, Massachusetts General Hospital, Charlestown, MA, USA; g Medical Physics Program, The University of Texas Graduate School of Biomedical Sciences, Houston, TX, USA

**Keywords:** Oropharynx, IMRT, Radiation, Toxicity, Presbyphagia

## Abstract

**Objectives:**

We sought to model chronic radiation-associated dysphagia (RAD) in patients given intensity-modulated radiation therapy (IMRT) for oropharyngeal squamous cell cancer (OPSCC) as a function of age and dose to non-target swallowing muscles.

**Methods:**

We reviewed 300 patients with T1-T4 N0-3 M0 OPSCC given definitive IMRT with concurrent chemotherapy. Chronic RAD was defined as aspiration or stricture on videoflouroscopy/endoscopy, gastrostomy tube, or aspiration pneumonia at ≥12 months after IMRT. Doses to autosegmented regions of interest (ROIs; inferior, middle and superior constrictors, anterior and posterior digastrics, mylo/geniohyoid complex, intrinsic tongue, and gengioglossus) were obtained from DICOM-RT plans and dose-volume histograms. The probability of chronic RAD as a function of mean ROI dose, stratified by age (<50, 50–59, 60–69, or ≥70 years), was estimated with logistic probability models and subsequent unsupervised nonlinear curves.

**Results:**

Chronic RAD was observed in 34 patients (11%). Age was a significant correlate of chronic RAD, both independently and with dose for all muscle groups examined. Distinct muscle-specific dose–response profiles were observed as a function of age (e.g., 5% of patients in their 50 s [but 20% of those 70 + ] who received 60 Gy to the superior constrictor had chronic RAD). This effect was stable across all observed muscle ROIs, with a false discovery rate-corrected *p* < 0.05, for all dose/muscle/age models, suggesting that including age as a covariate improves modeling of chronic RAD.

**Conclusions:**

Age at treatment moderates the probability of chronic RAD after chemo-IMRT for OPSCC, with aging muscles showing lower dose thresholds. Uniform dose constraints may not predict toxicity in older patients.

## Highlights

• Age at treatment for OPSCC is a strong predictor of chronic radiation associated dysphagia (RAD).
• For swallowing regions of interest (ROIs), dose to ROI and age impact patients’ risk of chronic RAD.
• For patients at high risk for RAD more intense prophylactic swallowing therapies may be warranted.

## 1 Introduction

Treatment outcomes after chemoradiation for head and neck cancer have improved, and patients are living longer in the HPV (human papillomavirus) era [1]. Given the growing population of patients with a high probability of survival, much attention surrounds late radiation-associated side effects. The morbidity of therapy is not to be taken lightly, as chronic long-term side effects can be devastating to patients’ health and quality of life [2], [3]. Chronic or late radiation-associated dysphagia (RAD) is among the most notable late complications of definitive chemoradiation [4], [5], [6], [7]. Accordingly, numerous research efforts have focused on risk reduction strategies for dysphagia, primarily dose optimization, proactive swallowing therapies, and pain management [2], [4], [5], [6], [8], [9], [10].

Chronic RAD is a dose and volume dependent toxicity. Non-target pharyngeal constrictors and the larynx are the classic regions of interest (ROIs) associated with swallowing. Numerous reports have shown that dose-volume variables associated with swallowing ROIs predict dysphagia [5], [11], [12]. Although classic normal tissue complication probability (NTCP) models take only dose into account, it is likely that patient-specific variables, such as age, also modulate the relationship between toxicity and dose to various ROIs.

We recently reported dose–response relationships highlighting the role of submental muscle dose (mylo/geniohyoid) in the development of chronic RAD among oropharyngeal cancer survivors. In this analysis, age at diagnosis was an important clinical characteristic correlated with prevalence of chronic RAD in multivariate models including dose [5], [11]. Building on this observation and those of many groups that have reported age at diagnosis as a predictor of chronic RAD, herein, we seek to explore this relationship further [2], [4], [13].

Presbyphagia, that is, age-related change in the physiology of swallowing, has been well documented in otherwise healthy aging individuals [14], [15], [16]. Swallowing is a submaximal effort activity meaning that only a portion of the maximal muscle capacity is used in a typical swallow. With age, however, the functional reserve a patient has to overcome new insults to swallowing function may be diminished. On this basis, we hypothesized that individual differences in prevalence of chronic RAD may be due to these underlying natural changes in swallow function with age [15], [16]. That is, age-related loss of functional reserve may make older patients more sensitive to radiation effects at a given dose. Moreover, existing presbyphagia likely reduces physiologic reserves to compensate for unavoidable functional loss after chemoradiation, and therefore aging patients may be inherently at higher risk for late radiation toxic effects [14]. For this reason, we evaluated potential differences in the dose-dependent predicted prevalence of chronic RAD as a function of age. To this end, we (1) defined the proportional effect of age as a covariate of dose-dependent chronic RAD, and (2) defined age-specific dose constraints to non-target swallowing ROIs to maintain the rates of predicted RAD at < 5%.

## 2 Materials and methods

We evaluated 300 patients in an existing, previously described cohort who received concurrent chemoradiation with intensity-modulated radiation therapy (IMRT) for oropharyngeal squamous cell carcinoma (OPSCC) at The University of Texas MD Anderson Cancer Center from 2002 through 2011 [11]. Inclusion criteria were: age ≥ 18 years, receipt of concurrent chemoradiation therapy with curative intent, pathologically confirmed OPSCC, available IMRT plans, bilateral neck treatment, and minimum follow-up time of ≥1 year after completion of radiation. This study was completed under an institutional review board–approved protocol. No patients with well lateralized tonsil tumors and ipsilateral nodal treatment were included.

Chronic RAD was chart abstracted, defined according to published criteria to include any of the following events at ≥1 year after radiation: aspiration or stricture (detected on videoflouroscopy or endoscopy), gastrostomy tube, or aspiration pneumonia [13]. Gastrostomy tube rates were coded at several time points (1-year follow-up, 2-year follow-up, and last disease-free follow-up). Videoflouroscopy or endoscopy were obtained upon referral of patients to a speech language pathologist (SLP) for symptoms of dysphagia; 69 such patients had some additional work up with SLP for concerning symptoms with imaging at ≥ 1 year after treatment.

**Fig. 1:**
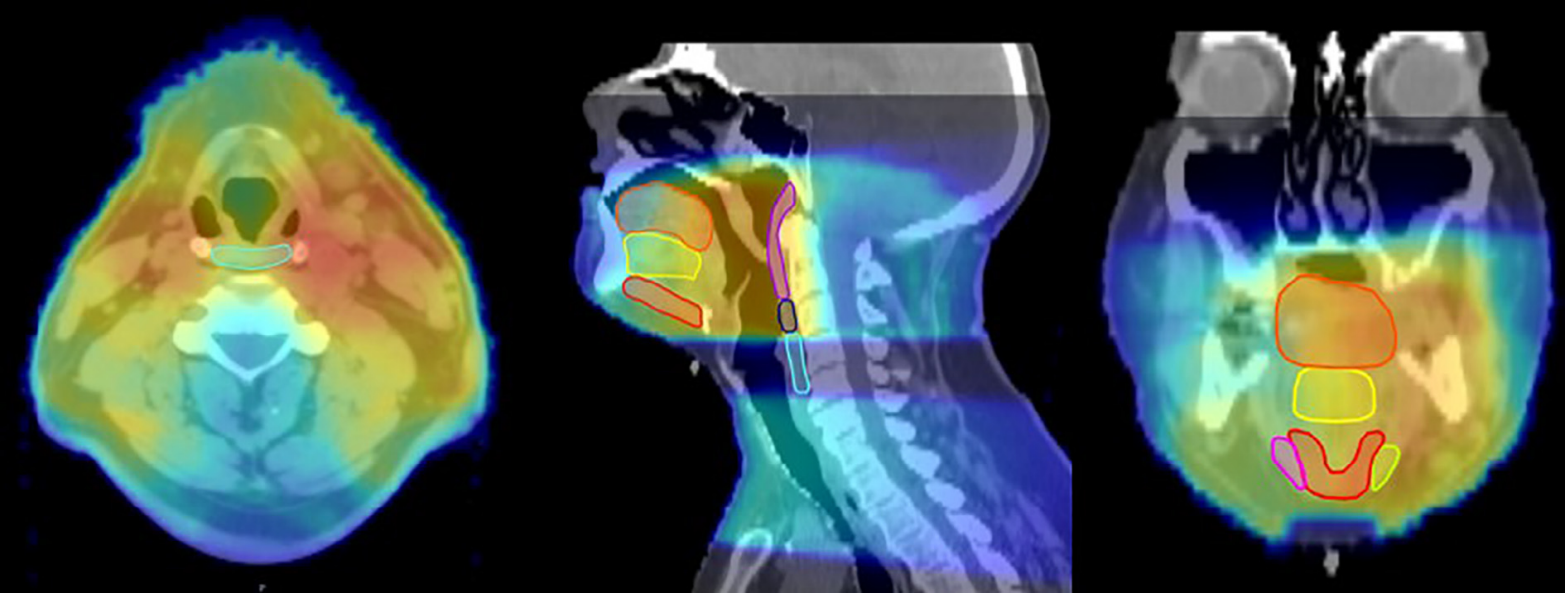
Example of auto-segmented ROIs on axial, sagittal, and coronal views with dose wash overlay. Muscle groups shown are the anterior digastric muscle (coronal, magenta and light green); genioglossus muscle (sagittal/coronal, yellow); inferior pharyngeal constrictor (sagittal/axial, aqua); intrinsic tongue muscle (sagittal/coronal, orange); mylo/geniohyoid muscle (sagittal/coronal, red); middle pharyngeal constrictor (sagittal, navy blue); and superior pharyngeal constrictor (sagittal, purple). (For interpretation of the references to color in this figure legend, the reader is referred to the web version of this article.)

Age, sex, ethnicity, American Joint Committee on Cancer disease stage, TNM classification, tumor subsite (tonsil, base of tongue, or other) smoking history (never smoker, former/<10 pack-years, current/greater than 10 pack-years), and chemotherapy regimen were collected as clinical variables. Treatment plans and dosimetric data were extracted with the Pinnacle 9.6 program (Phillips Medical Systems, Andover, MA). We used a benchmarked commercial deformable registration/segmentation program (Velocity AI 3.0.1, Velocity Medical Solutions, Atlanta, GA) [17] to export DICOM files from computed tomography treatment-planning scans for each patient. Two independent radiation oncologists (CDF and DIR) reviewed the autosegmented non-target swallowing muscular ROIs as described previously [17]. Segmented muscle-specific ROIs were the inferior, middle, and superior constrictors (IPC, MPC, and SPC); anterior digastric muscle (ADM); intrinsic tongue muscles (ITM); mylo/geniohyoid muscle complex (MGM); and genioglossus (GGM). Dose-volume histogram (DVH) information was available for all of the above ROIs. Representative sample ROIs and dose color-wash are shown in Fig. 1.

DVH data were used to generate a generalized mean dose calculation for each ROI as a way to account for spatial dose heterogeneity across the ROI volume. Dose was converted into 2 Gy-equivalents by using the biologically effective dose [BED] model to account for fractionation differentials; consequently, hereafter dose is reported in 2-Gy equivalents after normalization [18].

Individual dose/dysphagia NTCPs for the ROIs were fitted as 2-parameter models using the Wedenburg method [19]. The Bayesian information criteria (BIC) algorithm was used to select the covariates that would generate the most parsimonious model for predicting swallowing dysfunction. A regression model was constructed using the clinical variables described. The resultant model indicated mean dose and age as the most predictive covariates. The selected covariates were incorporated into an ordinal logistic model that was used to estimate the probability of dysphagia by age strata. The probability of dysphagia as a function of mean dose, stratified by age, was estimated by using logistic probability models and subsequent unsupervised nonlinear curves.

## 3 Results

### 3.1 Patient and treatment characteristics

Characteristics of the 300 patients included in this study are described in our paper by Dale et al. [11]. Median follow-up time from radiotherapy was 48 months, with at least 12 months of post-therapy surveillance (range 12–110 months). For the entire cohort, 82% (n = 247) of patients were stage T2-4 and 91% (n = 272) of patients had N2a-N3 nodal disease by AJCC 7th edition. The population was mostly male (n = 272, 91%) and Caucasian (n = 283, 94%). Concurrent Cisplatin was used in 195 patients (65%), with concurrent Cetuximab in 105 patients (35%). Median radiation dose was 70 Gy (range 64–75 Gy). Most patients (n = 262, 87%) were treated with standard once-daily fractionation.

When grouping patients by decade of life, there were 58 patients (19%) ≤49 years of age, 148 patients (49%) aged 50–59, 68 patients (23%) aged 60–69, and 26 patients (9%) aged ≥70 years.

### 3.2 Chronic radiation-associated dysphagia

According to the predefined criteria, 34 patients in this study (11%) had evidence of chronic RAD; 21 had videoflouroscopy-detected aspiration (7%), 10 had videoflouroscopy-detected stricture (3%), 18 had a gastrostomy tube at 12 months (6%), 10 had a tube at 24 months (3%), and 12 had a tube at last disease-free follow-up (4%). Overall 7% of patients who were ≤49 years old developed chronic RAD (n = 4), 9% of patients aged 50–59 (n = 14), 16% of patients aged 60–69 (n = 11), and 19% (n = 5) of patients aged ≥70 years. Clinical variables for patients showing symptoms of chronic RAD by decade of life are shown in Table 1**.**

**Table 1:** Clinical and treatment characteristics for patients with chronic radiation-induced dysphagia by decade of life.

	≤49 (n = 4)	50–59 (n = 14)	60–69 (n = 11)	≥70 (n = 5)
T3-4	3 (75)	10 (71)	6 (55)	2 (40)
N2b–3	4 (100)	13 (93)	10 (91)	5 (100)
Male sex	4 (100)	14 (100)	11 (100)	5 (100)
White race	4 (100)	13 (93)	11 (100)	5 (100)
Concurrent Cisplatin	4 (100)	14 (100)	10 (91)	5 (100)
Standard fractionation	3 (75)	14 (100)	8 (73)	4 (80)

AJCC 7th Edition Staging.

**Fig. 2:**
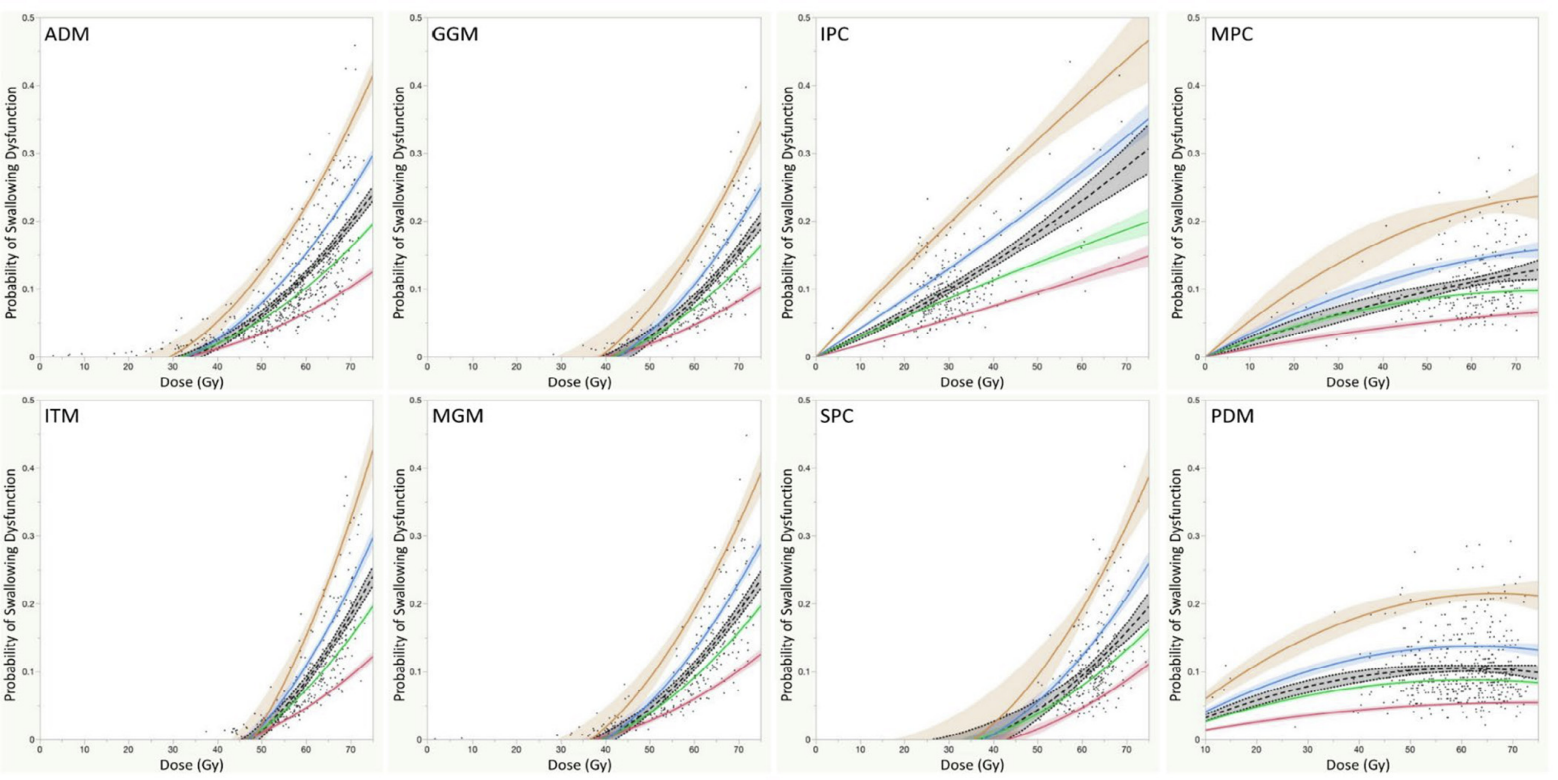
Two-parameter normal tissue complication probability curves for all regions of interest show significant interactions between age at treatment and development of chronic radiation-associated dysphagia at given dose levels. *Abbreviations*: NTCP, normal tissue complication probability; ROIs, regions of interest; ADM, anterior digastric muscle; GGM, genioglossus muscle; IPC, inferior pharyngeal constrictor; ITM, intrinsic tongue muscle; MGM, mylo/geniohyoid muscles; MPC, middle pharyngeal constrictor; PDM, posterior digastric muscle; SPC, superior pharyngeal constrictors. Legend – Orange, Age: ≥ 70– Blue, Age: 60–69– Green, Age: 50–59– Red, Age: ≤49 – Gray, All patients.

From our previous work, on univariate analysis age, T-category, N-category, gender, and cytotoxic chemotherapy showed significant differentials between rates of chronic-RAD (p < 0.05). A forward stepwise regression model using these parameters showed age as the most predictive clinical covariate. A bootstrapped 2-parameter (age, dose) fit of the NTCP for chronic RAD as a function of age and mean dose to non-target swallowing ROIs is shown in Fig. 2. The graphs show the fitted data with stratification by age in decades. Although the shape of the dose–response curve varied by muscle group ROI, in general, at a given dose level given to a non-target ROI, the prevalence of chronic dysphagia increased with age at treatment. All models were significant in discriminating the dose–dysphagia relationship when stratified by age (decade) for all examined ROIs (*p* < 0.05 for all models).

Table 2 shows the false discovery rate *p* values for dose and age in an effort to minimize the risk of reporting falsely significant findings due to multiple comparisons. The Bayesian information criteria and values for the area under the receiver operating characteristic curve are also shown to demonstrate the strength of the model for each muscle group.

**Table 2:** False discovery rate *p* values for dose and age for multiple comparisons.

Muscle	Dose false-discovery rate *p* value	Age false-discovery rate *p* value	Bayesian information criteria	Receiver operating characteristic AUC
Anterior digastric	0.0005	0.0443	176	0.7512
Genioglossus	0.0337	0.0337	184	0.7063
Inferior pharyngeal constrictor	0.1619	0.0369	187	0.661
Intrinsic tongue	0.0040	0.0366	180	0.736
Mylo/geniohyoid	0.0056	0.0358	180	0.729
Middle pharyngeal constrictor	0.9808	0.0243	189	0.649
Posterior digastric	0.7406	0.0224	189	0.6655
Superior pharyngeal constrictors	0.1192	0.0217	187	0.7136

*Abbreviation:* AUC, area under the curve.

## 4 Discussion

Chemoradiation for OPSCC carries a risk of late toxic effects such as chronic RAD [20]. Rates of clinically significant chronic dysphagia after conventional chemoradiation may be as high as 20%, with lower rates reported after more modern treatments such as IMRT or intensity-modulated proton therapy [4], [5], [6], [8], [21], [22]. With the recognition that tumors associated with human papillomavirus have a better prognosis, ongoing clinical trials are aiming to de-intensify therapy in efforts to mitigate late complications for patient subsets with favorable characteristics [10], [23]. Intensity-modulated radiation and de-intensification strategies rely on delivering less dose to non-target swallowing muscles to improve rates of dysphagia. Results from these ongoing trials will take years to mature, and as such it is important to continue to evaluate and model the normal tissue tolerance of adjacent non-target ROIs using standard doses.

There is a natural decline in swallowing function with increasing age, known as presbyphagia. Presbyphagia is distinct from dysphagia in that presbyphagia is considered healthy but involves age-related physiologic changes [24], [25], [26]. Various mechanisms contribute to these age-related changes in swallow function, including diminished lingual pressure, decreased salivary flow, sensory changes, pharyngeal atrophy, and sarcopenia [24], [27], [28]. These physiologic changes likely make older patients more susceptible to dysphagia from secondary insults, such as tumor and/or chemoradiation [29]. Thus, in the setting of head and neck cancer, an older patient with presbyphagia at baseline may have less reserve functionality to compensate for the stressors caused by the tumor and chemoradiation. In the present study we show that more aged swallowing structures are more sensitive to a given dose of radiation. Based on our findings, a given dose to certain swallowing ROIs can lead to a fourfold increase in the likelihood of late dysphagia based on the age of diagnosis. This may ultimately manifest as more common or more severe dysphagia among survivors, which in older patients can cause serious downstream effects such as dehydration, malnutrition, silent aspiration, pneumonia and non-cancer mortality [30], [31], [32], [33].

Classic NTCP models treat patient populations as uniform and monolithic with regard to response, and fail to account for patient-specific factors (such as age) or treatment factors (such as chemotherapy) as modifiers. Our findings and those of others underscore the need for multivariate models that incorporate both dose and clinical metrics in a more comprehensive way to aid in patient risk stratification and improve the therapeutic index of chemoradiation for OPSCC [11], [34], [35]. While factors such as T- and N-classification correlate with poorer swallowing (large tumor burden driving higher doses to larger volumes of non-target ROIs), advanced disease does not determine a patient’s individual sensitivity to a given radiation dose. The results of our study show the importance of considering other patient-specific or clinical factors, such as age, which might mitigate sensitivity in a ROI for a given dose level. The same may hold true for prior surgery and/or concurrent chemotherapy, both known to adversely impact swallowing outcomes and to be considered in future investigations.

The effect of age on long-term swallowing outcomes for patients with head and neck cancer treated with radiation has been noted in a few other studies [36], [37], [38], [39]. Generally speaking, clinical characteristics do influence the evaluation of dose constraints for individual patients, but incorporating clinical characteristics into standard normal tissue constraints is not common practice. It is becoming increasingly evident that dose to a variety of muscles involved in swallowing can be associated with the development of chronic RAD [11], [12], [13], [40], [41], [42]. If age further compounds this issue, as our results suggest, it would be reasonable to use age-specific dose constraints for swallowing ROIs to maximize an individual patient’s therapeutic index. Our findings lead us to propose dosimetric variables for the ideal mean dose to non-target ROIs that would result in a risk of chronic RAD of < 5% (Table 3). Some ROIs (such as the constrictor muscles) show more discrete differential dose levels by age, while others (intrinsic tongue muscles) show less variability. It is unclear why certain muscles may be more or less sensitive to radiotherapy doses as patients age and this area warrants further study and validation.

**Table 3:** Suggested mean doses, in Gy, for each segmented region of interest based on patient age at diagnosis, to maintain chronic radiation-associated dysphagia rate at <5%.

	Patient age at diagnosis and risk of radiation-associated dysphagia			
Muscles	≤49	50–59	60–69	≥70
Anterior digastric	55	49	45	40
Genioglossus	62	55	52	47
Inferior pharyngeal constrictor	26	17	10	<10
Intrinsic tongue	60	56	54	52
Mylo/geniohyoid	57	52	49	45
Middle pharyngeal constrictor	50	23	15	<10
Posterior digastric	60	22	13	<10
Superior pharyngeal constrictor	62	54	50	44

By using a model that can predict likelihood of dysphagia based on age and planned dose to non-target muscles, clinicians would have the means to predict which patients are at high risk of late swallowing complications. This would allow better counseling and perhaps faster referral to specialists for symptom management. If more restrictive dose constraints to swallowing structures cannot be met for an elderly patient, clinicians should consider more rigorous interventions during and after treatment to try to balance the increased risk of dysphagia. Proactive therapy with swallowing exercises has been studied as a possible means to reduce chronic dysphagia after chemoradiation [43], [44]. More intensive proactive intervention schedules may be indicated for older patients deemed to be at high risk of developing chronic RAD.

As is true for other DVH-driven studies, a limitation of the present series is the inability to capture sub-ROI volumetric data and effects resulting from loss of spatial data in the transition of 3-dimensional dose distributions to 2D DVH data. The lack of 3D data leads to the potential for confounding, as we cannot account for correlates regarding the location and proximity of tumor or nodal targets, which is what ultimately drives the dose to non-target ROIs. In the current study, we used previously benchmarked ROI segmentation and atlas work flow, but variations in ROI segmentation can functionally alter assessment of normal tissue complications and should be noted as a dependency.

Retrospective series are also at risk for multiple biases, including selection bias and observation (or information) bias. These weaknesses are notable, especially as information bias relates to the reporting of toxic effects and medical comorbidities. We do not have pre-treatment swallowing assessments on all patients, and are working on the assumption that the physiologic changes of presbyphagia affect our patients’ baseline swallowing as they age. Fortunately, we had excellent follow-up, with more than 94% of patients having at least 2 years of follow-up at the time of this analysis. Our institution uses clear guidelines for the multidisciplinary care and follow-up of patients with head and neck cancer. We essentially have a system in which any sign of dysphagia results in immediate referral to expert speech language pathologists. Institutional practice at the time of the study did not incorporate routine videoflouroscopy for patients without symptoms, which may have resulted in an artificially low rate of aspiration (essentially missing “silent” aspiration). Notably, however, the rate of chronic aspiration detected in this study mirrors the rate observed in an organ-preservation prospective study from MD Anderson Cancer Center during the same period in which videoflouroscopy was used routinely regardless of dysphagia symptoms [45].

The statistical methods used in the current study are somewhat distinct from typical NTCP approaches, insofar as the baseline probability of dysphagia is non-zero, and even at maximum therapeutic doses, dysphagia rates were observed in less than a majority for most cohorts. Consequently we implemented approaches involving 2-parameter curve with bootstrapping to preclude overfitting of the NTCP curve at higher generalized equivalent uniform dose (gEUD) levels.

Limitations aside, this study builds on our previous work, to date the largest study of patients with OPSCC treated with chemo-IMRT, by using benchmarked autosegmentation to investigate clinical and dosimetric correlates. Striking results herein find patient age at the time of radiation to be a distinct clinical factor that appears to correlate with rates of chronic RAD.

Our findings regarding differences in the rates of chronic RAD based on age at treatment could be immediately relevant in clinical practice. Although the dose to tumor is ultimately the main concern in treatment planning, our findings suggest that older patients should be counseled as to the risk of late dysphagia. Practitioners might use tighter dose-constraints for non-target ROIs based on the patient’s age. In light of our findings, for patients of advanced age, intensification of proactive swallowing therapy may be warranted to combat the higher risk of chronic RAD. Young patients who are given very high doses to non-target ROIs may also benefit from intensification of proactive therapy schedules. Future directions from this study would be to validate our findings by using autosegmentation of another group of patients with similar characteristics as the patients in this study. We further recommend the development of radiobiologic models that account for more than dose, to also include other clinical variables in an effort to move away from traditional dose-driven NTCP models, because dose response is not uniform across populations.

## 5 Conclusions

Age at treatment seems to moderate the dose–response probability of chronic RAD after chemo-IMRT for OPSCC. Our findings suggest that aging muscles show lower dose thresholds with regard to the development of potentially meaningful, clinically apparent late RAD. Further investigation of age-specific dose constraints and age-adjusted dysphagia prophylaxis models seems to be warranted. Our findings suggest that uniform monolithic dose constraints may fail to accurately predict or preclude clinical toxicity in older patients.

## 6 Funding statement

Research efforts of the MD Anderson Head and Neck Cancer Symptom Working Group^†^ were accomplished with infrastructure support of the multidisciplinary Stiefel Oropharyngeal Research Fund of The University of Texas MD Anderson Cancer Center Charles and Daneen Stiefel Center for Head and Neck Cancer. The Spatial-Non-spatial Multi-Dimensional Analysis of Radiotherapy Treatment/Toxicity Team^§^ (SMART_3,_) is supported by a multi-site National Science Foundation (NSF), Division of Mathematical Sciences, Joint NIH/NSF Initiative on Quantitative Approaches to Biomedical Big Data (QuBBD) Grant (NSF 1557679) and the NIH Big Data to Knowledge (BD2K) Program of the National Cancer Institute (NCI) Early Stage Development of Technologies in Biomedical Computing, Informatics, and Big Data Science Award (1R01CA214825). Dr. Gunn received a philanthropic gift from the Family of Paul W. Beach, partially supporting the salaries of Drs. Jomaa and Elhalawani. Drs. Lai, Hutcheson, Mohamed and Fuller, as members of the MD Anderson Head and Neck Cancer Symptom Working Group^†^, received funding support from the National Institutes of Health (NIH)/National Institute for Dental and Craniofacial Research (1R01DE025248/R56DE025248), NIH/NCI (R21CA226200), and the NIH/NCI Early Phase Clinical Trials in Imaging and Image-Guided Interventions Program (1R01CA218148). Dr. Fuller receive(s/d) grant and/or efforts/salary support from the following entities/mechanisms/funders during the term of this research effort: the Andrew Sabin Family Foundation; The Mike Hogg Foundation; National Institute of Biomedical Imaging and Bioengineering (NIBIB) Research Education Programs for Residents and Clinical Fellows Grant (R25EB025787-01); an NIH/NCI Cancer Center Support Grant (CCSG) Pilot Research Program Award from the UT MD Anderson CCSG Radiation Oncology and Cancer Imaging Program (P30CA016672); the NIH/National Cancer Institute Head and Neck Specialized Programs of Research Excellence Developmental Research Program Award (P50CA097007); a Paul Calabresi Clinical Oncology Program Award (K12 CA088084); a General Electric Healthcare/MD Anderson Center for Advanced Biomedical Imaging In-Kind Award; an Elekta AB/MD Anderson Department of Radiation Oncology Seed Grant; the Center for Radiation Oncology Research at MD Anderson Cancer Center Seed Grant; and the MD Anderson Institutional Research Grant (IRG) Program.

## Declaration of Competing Interest

Dr. Frank reports personal fees from Varian, grants and personal fees from C4 Imaging, grants from Eli Lilly, grants from Elekta, grants and personal fees from Hitachi, other from Breakthrough Chronic Care, personal fees from Augmenix, and personal fees from National Comprehensive Cancer Center (NCCN), outside the submitted work. Dr. Phan reports other from Accuray, outside the submitted work. Dr. Rosenthal reports other from Merck, outside the submitted work. Dr. Fuller has received speaker honoraria, grant support, and travel funding from Elekta AB, outside of this work. Dr. Fuller has received speaker honoraria and/or travel funding from the Gilbert H. Fletcher Society/Tianjin Cancer Center Symposia, American Association of Physicists in Medicine, Greater Baltimore Medical Center, University of Illinois Chicago, The University of Texas Health Science Center at San Antonio, Oregon Health and Science University, and The Translational Research Institute Australia outside of this work. Dr. Fuller receives royalties from Demos Medical Publishing for publications outside of this work.
